# ALPK1 regulates streptozotocin‐induced nephropathy through CCL2 and CCL5 expressions

**DOI:** 10.1111/jcmm.14643

**Published:** 2019-09-26

**Authors:** Chi‐Pin Lee, Srinivasan Nithiyanantham, Hui‐Ting Hsu, Kun‐Tu Yeh, Tzer‐Min Kuo, Ying‐Chin Ko

**Affiliations:** ^1^ Environment‐Omics‐Disease Research Center China Medical University Hospital China Medical University Taichung Taiwan; ^2^ Department of Surgical Pathology Changhua Christian Hospital Changhua Taiwan; ^3^ Department of Pathology Changhua Christian Hospital Changhua Taiwan

**Keywords:** ALPK1, CCL2, CCL5, chemokine, diabetic nephropathy

## Abstract

ALPK1 is associated with chronic kidney disease, gout and type 2 diabetes mellitus. Raised renal ALPK1 level in patients with diabetes was reported. Accelerated fibrotic nephropathies were observed in hyperglycaemic mice with up‐regulated ALPK1. The aim of this study was to identify the mediators contributing to ALPK1 effect involving in nephropathies induction. The haematoxylin and eosin staining, Masson's trichrome and immunohistochemical analysis of ALPK1, NFkB, CCL2 and CCL5 were performed in the mice kidney. Cytokine antibody array analysis was performed in streptozotocin‐treated wild‐type mice (WT‐STZ) and streptozotocin‐treated ALPK1 transgenic mice (TG‐STZ). The ALPK1 levels were measured in mice kidney and in cultured cells. We found that the higher levels of renal CCL2/MCP‐1, CCL5/Rantes and G‐CSF expression in TG‐STZ compared with the WT‐STZ. Glucose increased ALPK1 expressions in monocytic THP1 and human kidney‐2 cells. The protein expression of ALPK1, NFkB and lectin was up‐regulated in glucose‐treated HK‐2 cells. Knockdown of ALPK1 reduced CCL2 and CCL5 mRNA levels, whereas overexpressed ALPK1 increased CCL2 and CCL5 in cultured kidney cells. Taken together, these results show that high glucose increases ALPK1 and chemokine levels in the kidney. Elevated ALPK1 expression enhances renal CCL2 and CCL5 expressions in vivo and in vitro. ALPK1 is a mediator for CCL2 and CCL5 chemokine up‐regulation involving in diabetic nephropathies induction.

## 1 INTRODUCTION

Alpha‐kinase 1 (ALPK1), a member of the alpha‐kinase family, has been reported to be highly associated with cancer and chronic diseases such as chronic kidney disease (CKD), type 2 diabetes mellitus (type 2 DM) and gout.^1^, ^2^, ^3^ ALPK1 mutant has been reported to cause severe defects in motor co‐ordination in mice.^4^ Mice with overexpression of ALPK1 exhibited lower levels of testosterone and urate/anion exchanger URAT1.^5^, ^6^ ALPK1 is also known to be involved in the phosphorylation of myosin IA and myosin IIA, which might participate in apical protein transport and TNF‐α secretion.^7^, ^8^ ALPK1 enhances the expression of proinflammatory cytokines including IL‐1β, IL‐8 and TGF‐β1 in cultured human THP1 and human embryonic kidney 293 (HEK293) cells.^9^, ^10^ ALPK1 controls the formation of the TIFA complex, which acts in a sequential manner to activate the transcription factor nuclear factor‐kappaB (NFkB) and regulate the production of IL‐8 in response to infections with *Shigella flexneri*, *Salmonella typhimurium*, *Neisseria meningitides* and *Helicobacter pylori*.^11^, ^12^ These observations suggest a master regulatory role of ALPK1 in inflammation and innate immunity.

Kidney damage is one of the more common long‐term complications of chronic diseases such as diabetes and gout. Recently, accumulated evidence has suggested diabetic nephropathy (DN) as an inflammatory disease.^13^ DN, which is a common leading cause of end‐stage kidney failure worldwide, is characterized clinically by proteinuria and pathologically by excessive deposition of extracellular matrix (ECM) proteins and glomerulosclerosis and tubulointerstitial fibrosis.^14^ NFkB that is activated under hyperglycaemic conditions plays a prominent role in the pathogenesis of DN, regulating the production of proinflammatory cytokines, chemokines and intercellular adhesion molecule 1 (ICAM1).^15^ The proinflammatory cytokines and chemokines are known to play a critical role in inflammation, enhancing the ECM synthesis, and renal fibrosis in renal injury.^16^, ^17^, ^18^ Patients with diabetic glomerulosclerosis have been reported to exhibit enhanced ALPK1 expression in the renal tubular epithelial cells.^2^ In addition, streptozotocin (STZ)‐treated mice with ALPK1 overexpression exclusively showed severe hyperglycaemia and accelerated multiple early nephropathies in the kidney.^10^ Based on this evidence obtained from bacterial‐infected cell model, diabetic animal model and human studies of chronic diseases, ALPK1 has been proposed as a central player in the inflammatory response.

Monosodium urate (MSU) crystal is an inducer of inflammation whose deposition in articular and periarticular tissues is the common feature of acute gouty arthritis. We have demonstrated that MSU crystal can increase the levels of ALPK1 in cultured cells and is involved in the induction of proinflammatory cytokines, including IL‐1β, IL‐8, TGF‐β1 and TNF‐α.^9^, ^10^ In contrast, testosterone was found to induce a repressive effect on the production of proinflammatory cytokines upon ALPK1 inhibition.^5^

The aim of this study was to analyse the mediators contributing to the effect of ALPK1 in the inflammatory response causing renal injury. We detected a correlation between the expression levels of ALPK1 and NFkB in the same renal tubular cells of the mouse. Increased ALPK1 expression enhanced the expression levels of renal monocyte chemoattractant protein‐1 (MCP‐1/CCL2) and RANTES/CCL5 (RANTES, an acronym for ‘Regulated upon Activation, Normal T cell Expressed and Secreted’) both in vivo and in vitro. Further, we also discuss the regulation of chemokine by ALPK1 under high‐glucose condition and its importance in relation to renal inflammatory response.

## 2 MATERIALS AND METHODS

### 2.1 Animal procedure

In this study, animal procedures conformed to the guidelines published by the National Institute of Health (NIH Publication No. 85‐23) and were approved by the Institutional Animal Care and Use Committee (IACUC) of the China Medical University. TG mice development procedure has been mentioned in our previous paper.^5^ Wild‐type C57BL/6 mice were purchased from the National Laboratory Animal Center (NLAC, Taipei, Taiwan).Eight‐week‐old animals are used in our study. The mice group details are, C57BL/6 male animals (WT), hALPK1 transgenic mice (TG), streptozotocin (STZ)‐treated C57BL/6 mice (WT‐STZ) and streptozotocin‐treated hALPK1 transgenic mice (TG‐STZ). The diabetic condition was induced by five consecutive intraperitoneal injections of low‐dose STZ (40 mg/kg, dissolved in ice and 0.1 mol/L citrate buffer) daily. The treatment period is for about 3 weeks after STZ injection.^10^ After treatment, all animals were sacrificed and measured ALPK1 levels and cytokine transcripts by RT‐qPCR for kidney tissue.

### 2.2 Assessment of cytokine secretion from the mice kidneys

Whole kidney lysates were collected from WT‐STZ and TG‐STZ. Measurement of renal cytokines and chemokines for comparative analysis was performed with a Bio‐Plex Pro™ mouse cytokine 23‐plex assay kit (Bio‐Rad).

### 2.3 Immunohistochemistry analysis

The renal tissue samples from all animals were fixed with 4% paraformaldehyde for 3 days and embedded in paraffin. The 5 μm sections were stained with haematoxylin and eosin (H&E), specific antibodies and Masson's trichrome for histological evaluation and scoring. The protein signal was detected using the appropriate primary antibody amplifier, horseradish peroxidase (HRP)‐conjugated polymer and DAB chromogen/substrate. The slides were immunostained with anti‐NFkB p65 (50‐fold of dilution; Abcam), anti‐ALPK1 (100‐fold of dilution; GeneTex Inc), anti‐CCL2 (50‐fold of dilution; Abcam) and anti‐CCL5 (50‐fold of dilution; Abcam). The tubular injury was scored based on the degree of tubular necrosis, loss of brush border, cast formation and tubular dilatation, as in previous reports.^19^, ^20^ The scoring standard was as follows: 0‐normal kidney; 1‐minimal injury (<5% involvement); 2‐mild injury (5%‐25% involvement); 3‐moderate injury (25%‐75% involvement); and 4‐severe injury (>75% involvement).

### 2.4 Cell culture and transfection

Human kidney‐2 (HK‐2, normal proximal tubule cells), HEK293 and human monocytic leukaemia THP1 cells were obtained and maintained. In this study, the authentication of cell lines has been performed by STR analysis. Mouse primary kidney cell was isolated from male wild‐type (C57BL/6) mice. The ALPK1 plasmid was subcloned from pSportSfi vector containing ALPK1 cDNA. ALPK1 cDNA was amplified using the specific primers and ligated into the unique restriction sites, BglII/SalI, of the pEGFP‐C1 vector (Clontech). The resulting plasmid denoted pEGFP‐ALPK1‐C1 was confirmed by sequencing. Used human ALPK1 or non‐specific control siRNAs were purchased from Invitrogen (Stealth siRNA, Invitrogen). For examination of ALPK1 effects on CCL2 and CCL5 expressions, plasmid DNA or siRNA was transfected into cells using the Lipofetamine™ 2000 (Invitrogen) transfection reagents according to the manufacturer's protocol. After 48 hours of transfection, the cells were harvested for further analysis. The mRNA levels of ALPK1, CCL2 and CCL5 transcripts were measured by RT‐qPCR.

### 2.5 Real‐time quantitative PCR (RT‐qPCR)

Total RNA was isolated from treated cells and tissues using TRIzol reagent according to the manufacturer's protocol (Invitrogen, USA). The cDNA templates were produced by reverse transcription of the total RNA with random primer and a high capacity cDNA reverse transcription kit (Applied Biosystems). The qPCR analysis with specific primer pairs was performed with Power SYBR^®^ Green PCR Master Mix (Life Technologies).

### 2.6 Western blotting

For detection of ALPK1, the cells were lysed with RIPA buffer containing protease inhibitors on ice for 10 minutes. The extracts were centrifuged at 14 500  g for 5 minutes at 4°C. Protein samples were separated by SDS‐PAGE gel electrophoresis and transferred to polyvinylidene fluoride membranes. The membranes were incubated with primary antibodies as indicated and peroxidase‐conjugated secondary antibodies and protein signals detected by enhanced chemiluminescence reagent. The primary antibodies used were as follows: anti‐ALPK1 (1000‐fold of dilution; GeneTex Inc), anti‐lectin (1000‐fold of dilution; Bioorbyt), anti‐NFkB (1000‐fold of dilution; Abcam) and anti‐Actin (3000‐fold of dilution; GeneTex Inc).

### 2.7 Immunofluorescence staining

HK‐2 cells were treated with high glucose (200 mg/dL) for 48 hours. After the treatment, cells were fixed with 4% paraformaldehyde for 15 minutes at room temperature and permeabilized with 0.5% triton X‐100 for 10 minutes at room temperature and then blocking for 1 hour. Add specific primary antibody and incubate at 4°C for overnight. Subsequently, the cells were washed and stained with Alexa 546 rabbit antimouse IgG secondary antibodies (Invitrogen) for 1 hour at room temperature. The cells were washed before nuclear staining with 1 μg/mL. Images were observed under a fluorescence microscope. The cell fluorescent intensity was measured using ImageJ software.

### 2.8 Statistical analysis

Quantitative data of mouse cytokines and chemokines levels were compared between the WT‐STZ and TG‐STZ using the Wilcoxon rank‐sum test. All data were expressed as mean ± standard deviation. Quantitative data were performed using unpaired Student's *t* test. Then, for cell culture experiments the statistical differences were evaluated using Student's *t* test. The level of significance was *P < .05, **P < .01, ***P < .001, ****P < .0001.

## 3 RESULTS

### 3.1 ALPK1 regulates STZ‐induced nephropathy through chemokine in transgenic mice

**Figure 1:**
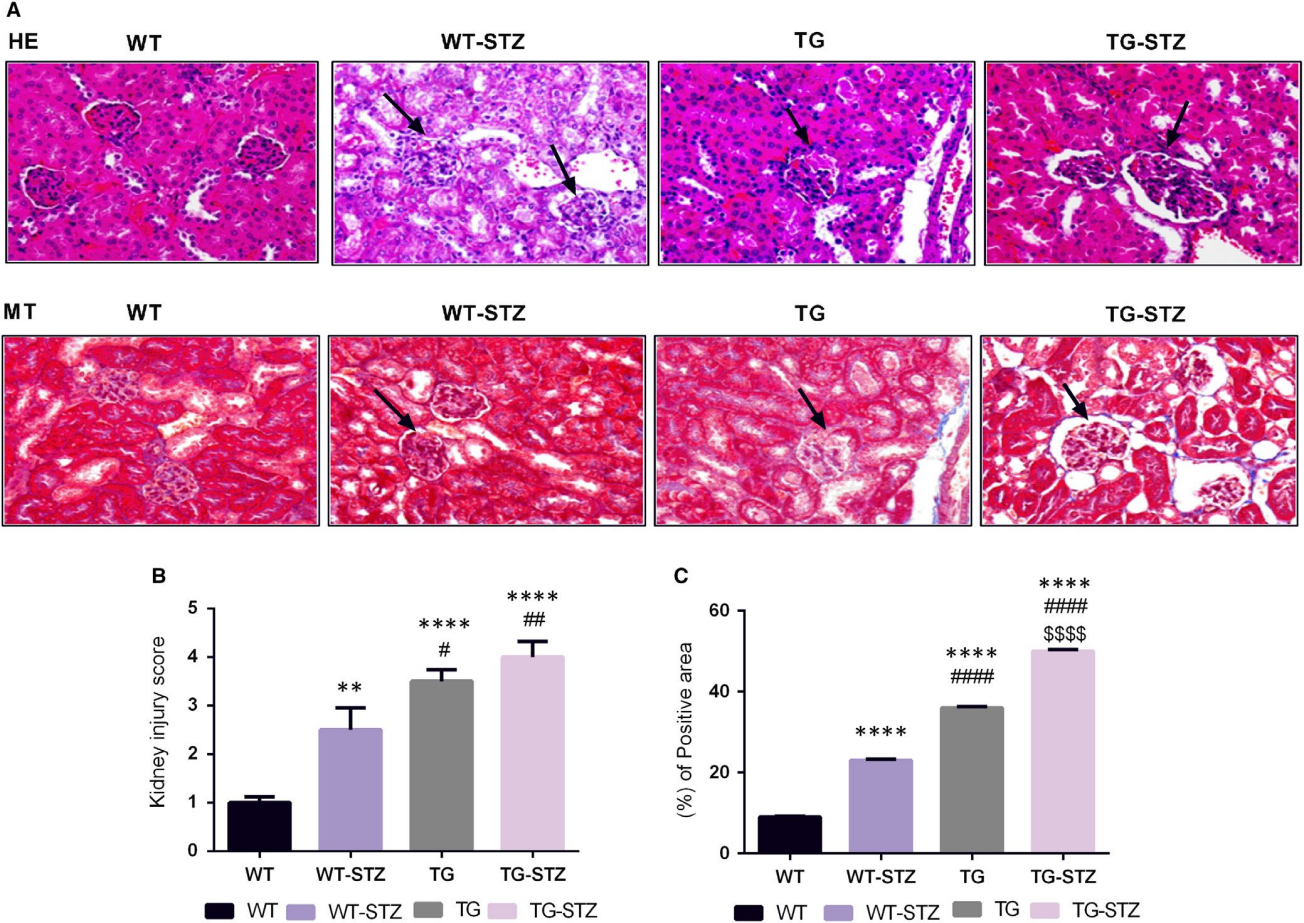
Histological examinations of kidney tissues from WT, WT‐STZ, TG and TG‐STZ (A). Haematoxylin and eosin staining and Masson's trichrome staining (B). Kidney injury score of four groups (C). Collagen deposition was higher in TG‐STZ. **P < .01 are compared to the WT; ****P < .0001 are compared to the WT; #*P* < .05 are compared to the WT‐STZ; ##*P* < .01 are compared to the WT‐STZ; ####*P* < .0001 are compared to the WT‐STZ; and $$$$*P* < .0001 are compared to the TG

**Figure 2:**
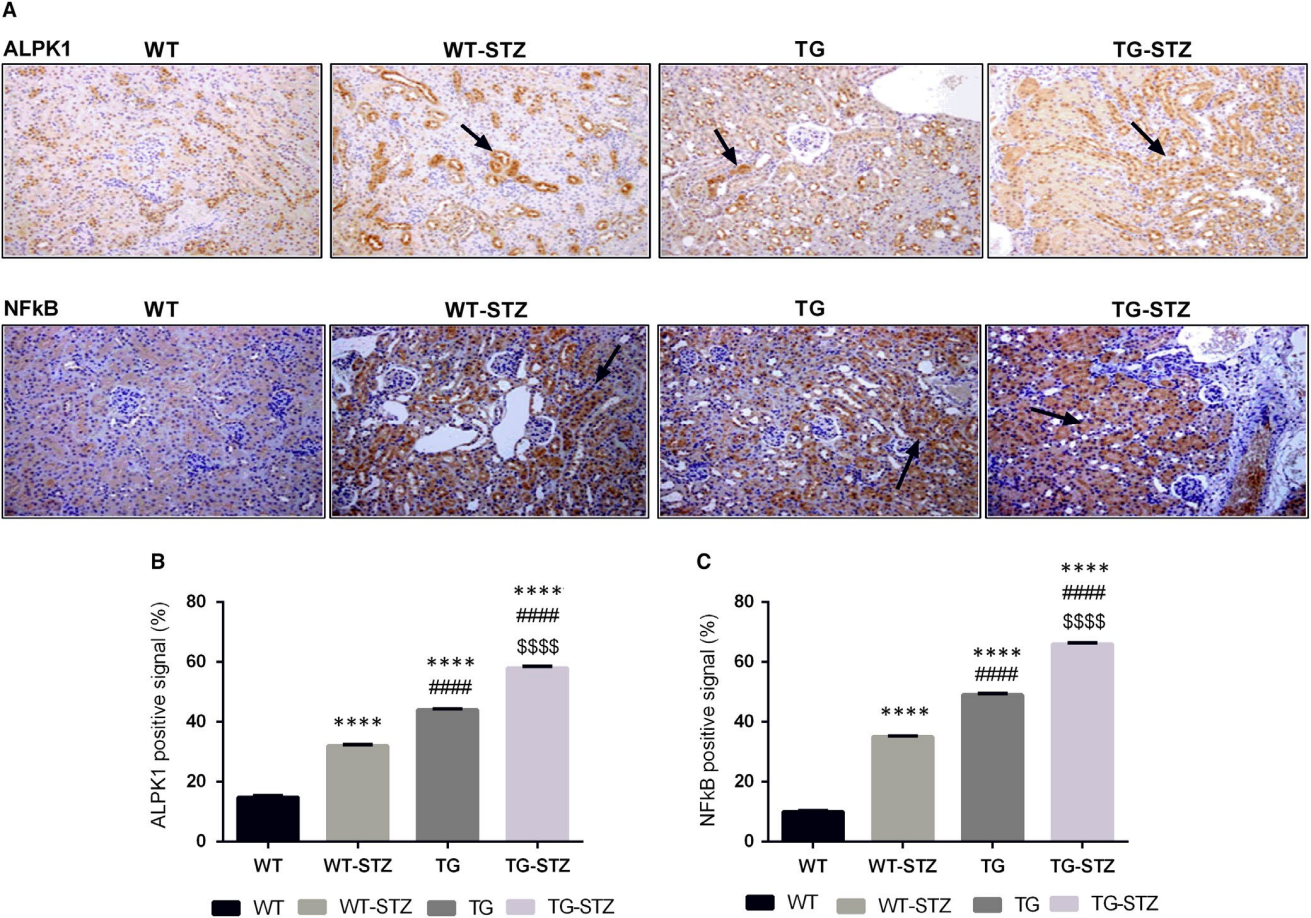
Immunohistochemical expression of ALPK1 and NFkB (A). The up‐regulation of ALPK1 and NFkB expression in STZ treated mice and overexpression of ALPK1 mice. Brown positive signals were predominantly distributed in renal tissues (B, C). ALPK1 and NFkB positive cells in renal tissue from the immunohistochemical analysis of WT, WT‐STZ, TG and TG‐STZ. These graphs represent mean ± SD. ****P < .0001 are compared to the WT; ####*P* < .0001 are compared to the WT‐STZ; and $$$$*P* < .0001 are compared to the TG

**Figure 3:**
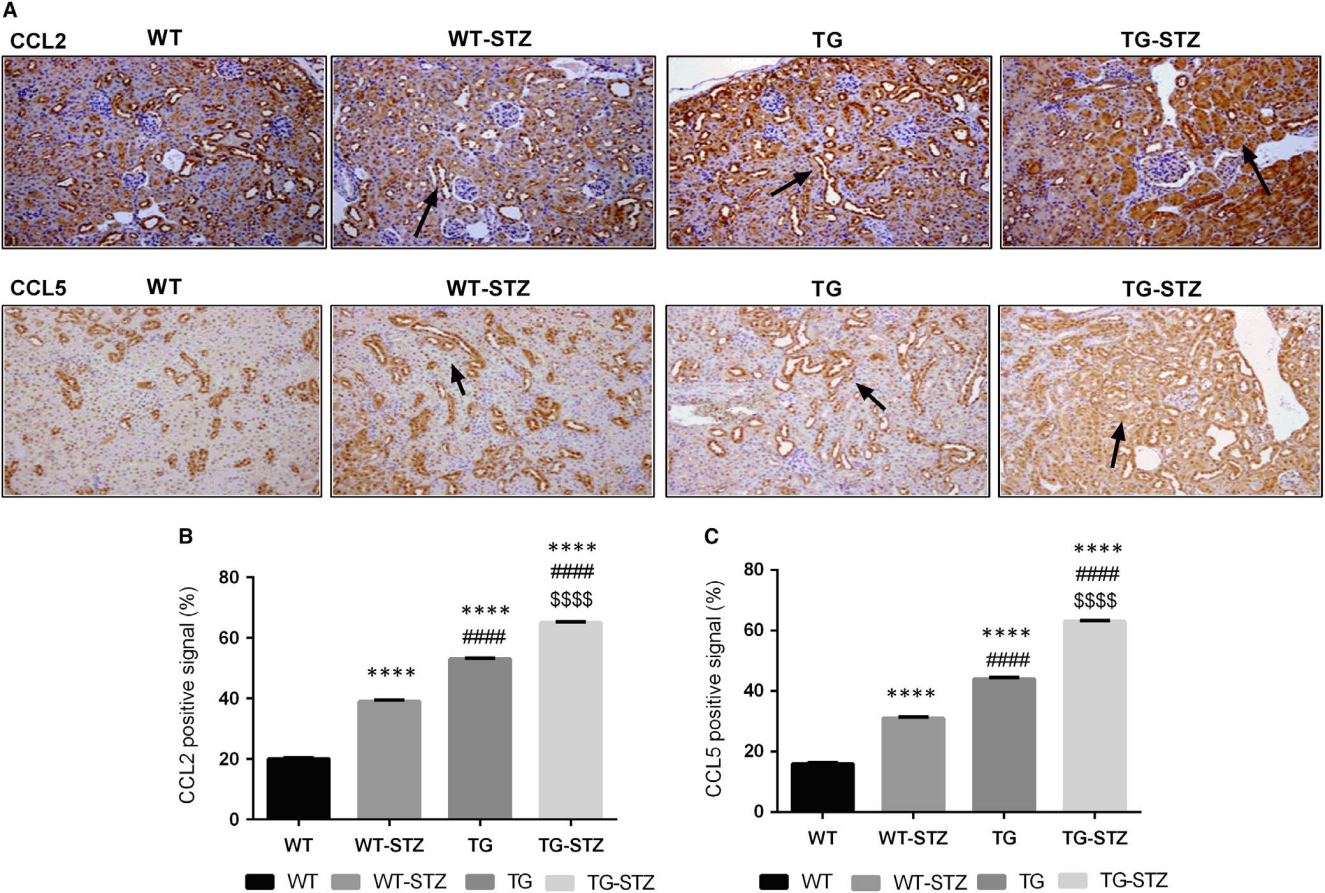
Immunohistochemical expression of CCL2 and CCL5 (A). The up‐regulation of CCL2 and CCL5 expression in STZ‐treated mice and overexpression of ALPK1 mice. Brown positive signals were predominantly distributed in renal tissues (B, C). CCL2 and CCL5 positive cells in renal tissue from the immunohistochemical analysis of WT, WT‐STZ, TG and TG‐STZ. These graphs represent mean ± SD. ****P < .0001 are compared to the WT; ####*P* < .0001 are compared to the WT‐STZ; $$$$*P* < .0001 are compared to the TG

The TG‐STZ mice were employed for investigating the role of ALPK1 in diabetic nephropathy pathogenesis. Hyperglycaemia was found in both wild‐type and hALPK1‐TG mice after STZ injection. The microscopic examination of haematoxylin‐and‐eosin (H&E)–stained section reveals higher tubular cell necrosis, cytoplasmic vacuoles, loss of brush border and tubular dilation in TG and TG‐STZ mice compared with the WT mice (*P* < .0001) (Figure 1). Additionally, TG‐STZ mice showed higher collagen deposition compared with the WT mice significantly (*P* < .0001). Then, we found a higher significant difference between TG and TG‐STZ (*P* < .0001), WT and WT‐STZ (*P* < .0001), and WT‐STZ and TG‐STZ (*P* < .0001). Nonetheless, abundant ALPK1 and NFkB expressions were detected in renal tubular cells significantly (Figure 2). In addition, the chemokine CCL2 and CCL5 expressions were up‐regulated in WT‐STZ, TG and TG‐STZ compared with the control groups significantly (*P* < .0001) (Figure 3). Overall, the expression of target proteins was up‐regulated in TG‐STZ compared with the control groups.

### 3.2 Effects of ALPK1 on renal cytokine/chemokine release in STZ stimulated mice

**Figure 4:**
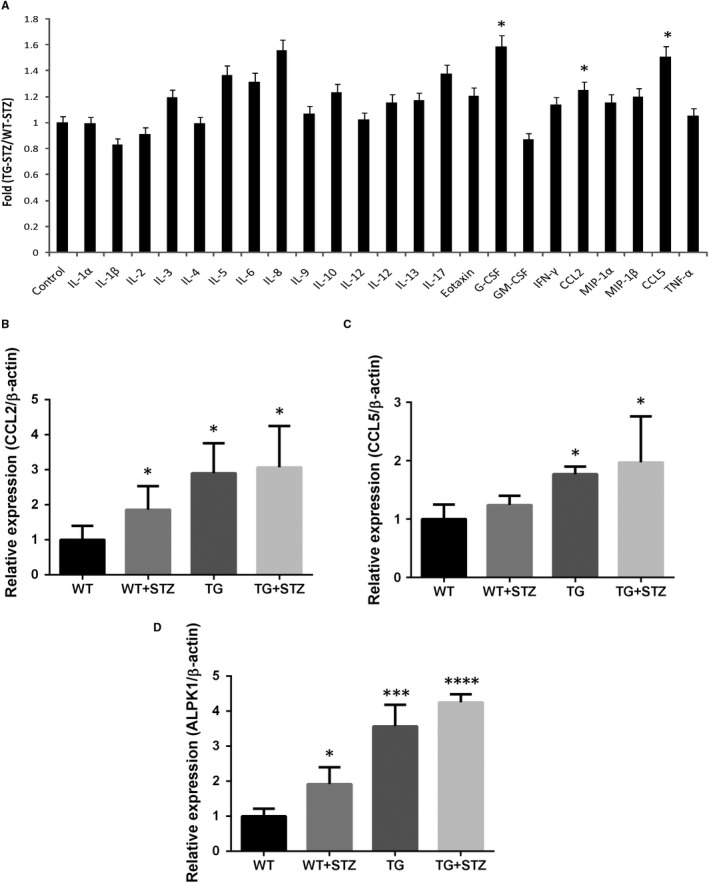
Cytokine and chemokine secretion increased in TG‐STZ mice (A). Bio‐Plex Pro™ mouse cytokine array was used to screen cytokine or chemokine secretion in kidney samples from WT‐STZ and TG‐STZ (B‐D). Transcripts of CCL2, CCL5 and ALPK1 were measured by RT‐qPCR. Results of RT‐qPCR were repeated three times. Higher ALPK1 levels correlate with increased cytokine and chemokine expression in STZ‐treated mice. Values shown in the graph are mean ± SD. *P < .05 are compared with the WT; ***P < .001 are compared with the WT; ****P < .0001 are compared with the WT

Then, a selected 23 cytokines/chemokines antibody array analysis on the kidney lysate from TG‐STZ and WT‐STZ mice was performed. As shown in Figure 4A, TG‐STZ mice had significantly higher granulocyte colony‐stimulating factor (G‐CSF) (1.59‐fold, *P* = .023), MCP‐1/CCL2 (1.25‐fold, *P* = .046) and RANTES/CCL5 (1.51‐fold, *P* = .023) than WT‐STZ. Additionally, keratinocyte‐derived cytokine (KC/CXCL1/IL‐8) trended to up‐regulation in the TG‐STZ group compared with the WT‐STZ group (1.56‐fold, *P* = .064). These results suggest that the elevated ALPK1 expression enhances the renal levels of cytokine/chemokine protein in mice with a hyperglycaemic condition.

### 3.3 Higher renal levels of CCL2 and CCL5 mRNA expression in transgenic mice

We had compared the renal mRNA levels of ALPK1, CCL2, CCL5, G‐CSF and IL‐8 for all four groups. Higher mRNA levels of CCL2 and CCL5 in TG‐STZ mice were compared with the control mice (*P* < .05) (Figure 4B‐D). Nonetheless, we found significant lower levels of G‐CSF transcripts in TG mice compared with the control mice. In addition, there is no significant difference among the groups in IL‐8 transcripts. These results show that higher ALPK1 level correlates with higher levels of CCL2 and CCL5, indicating that ALPK1 may enhance the production of CCL2 and CCL5 in mice.

### 3.4 The up‐regulation of ALPK1 in cultured kidney cells by glucose stimulation

**Figure 5:**
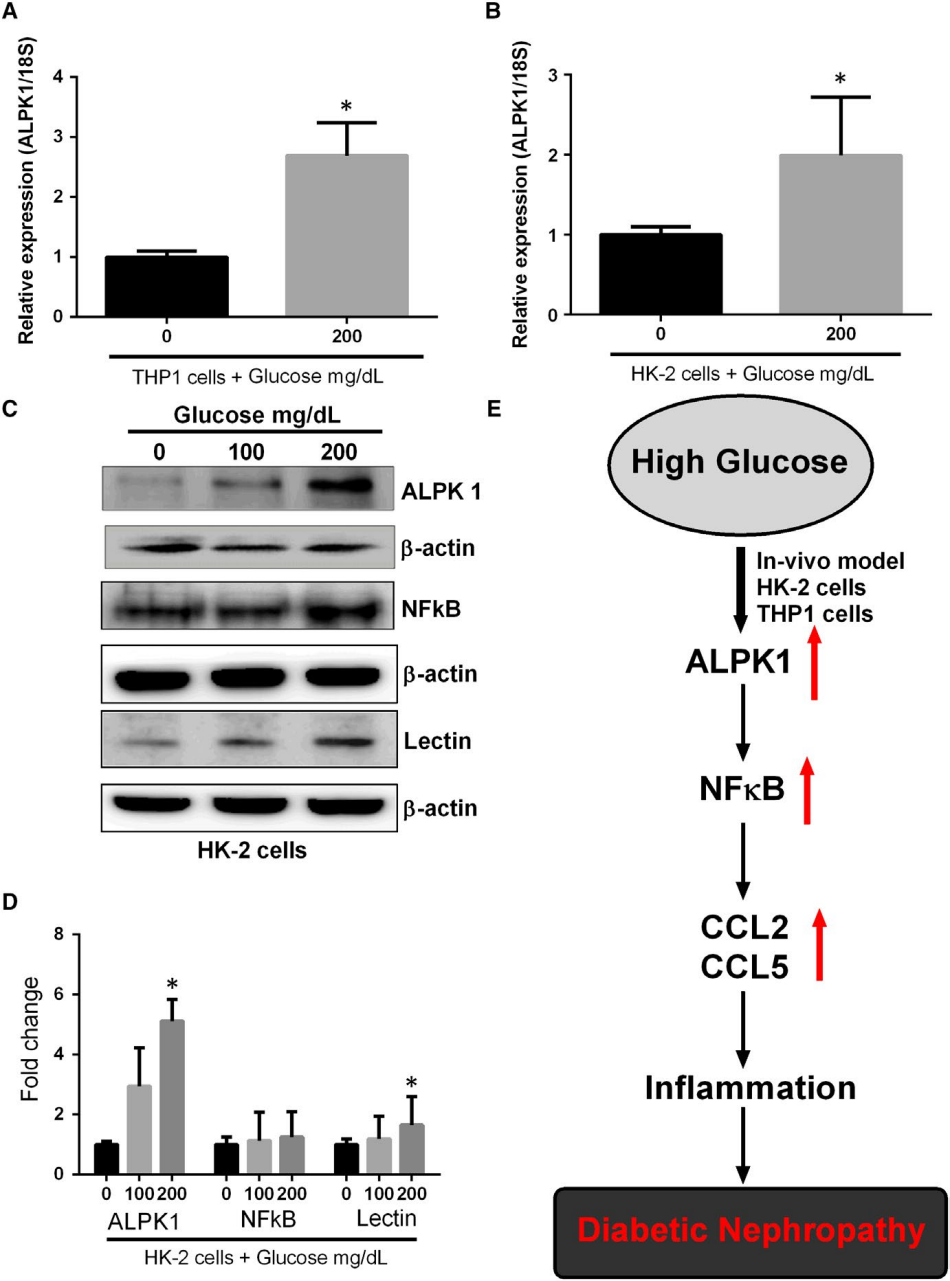
Up‐regulation of ALPK1 in cultured kidney cells by glucose stimulation. THP1 and HK‐2 cells were treated with glucose for 48 h. Transcripts of ALPK1 were measured by RT‐qPCR. Glucose increased ALPK1 mRNA levels in (A). THP1 (B). HK‐2 cells. Graphs represent mean ± SD values, and each experiment was performed in triplicate (C). The protein expression of ALPK1, NFkB, lectin and β‐actin was determined by Western blotting in HK‐2 cells (D). The enhanced effect of glucose on ALPK1, NFkB and lectin protein expressions was plotted on the bar graph. ALPK1 protein signal was quantified by densitometry analysis and expressed as a fold change in respective control cells from three independent experiments (E). Schematic model showing that the ALPK1 regulates glucose induced nephropathy through CCL2 and CCL5 expressions. ∗*P* < .05 compared with the THP1 or HK‐2 cells without glucose stimulation

**Figure 6:**
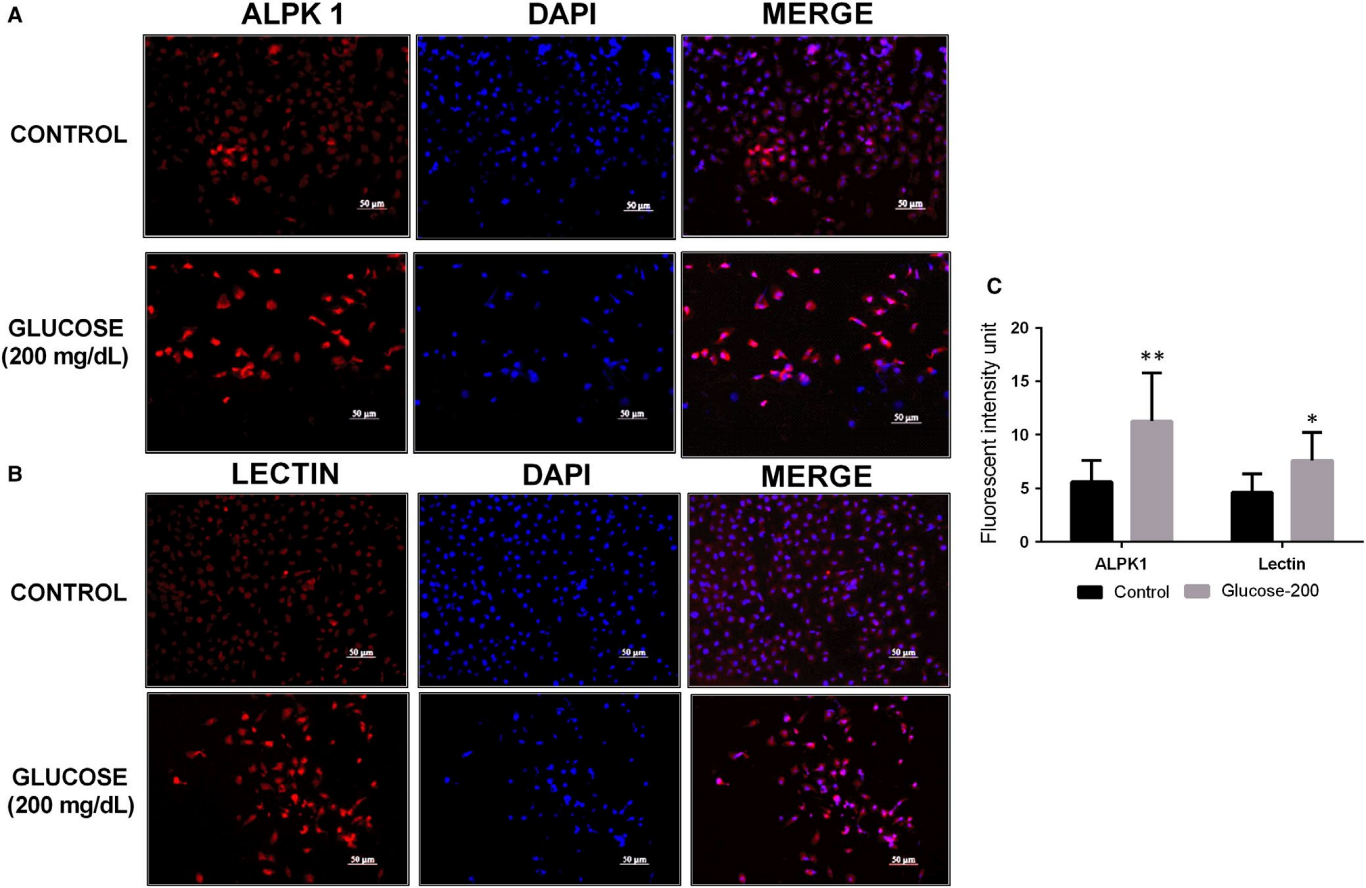
ALPK1 and lectin expression in HK‐2 cells. (A, B). Immunofluorescence staining showed a high level of ALPK1 and lectin expressions in HK‐2 cells treated with high glucose. Cell nuclei were stained with DAPI (Blue). (C). Quantification of cell fluorescent intensity. *P < .05 are compared with the control cells; **P < .01 are compared with the control cells

The glucose effects on ALPK1 induction in THP1 cells were evaluated using RT‐qPCR. Glucose stimulation significantly increased the levels of ALPK1 transcripts (Figure 5A). Then, we investigated whether glucose increased ALPK1 expression in cultured kidney cells. Confluent human kidney HK‐2 cells were cultured in the medium with raised glucose levels for 48 hours. Levels of ALPK1 mRNA and protein were measured. Glucose significantly increased both ALPK1 mRNA and protein levels (*P* < .05) (Figure 5B‐D). Then, the protein expression of NFkB and lectins (*P* < .05) was up‐regulated in glucose‐treated HK‐2 cells. Immunofluorescence staining showed a high level of ALPK1 (*P* < .01) and lectin (*P* < .05) expressions in glucose‐treated HK‐2 cells compared with the control cells (Figure 6). These results suggest that glucose enhances ALPK1 expression in cultured THP1 and HK‐2 cells.

### 3.5 Overexpression and knockdown of ALPK1 in cultured kidney cells

**Figure 7:**
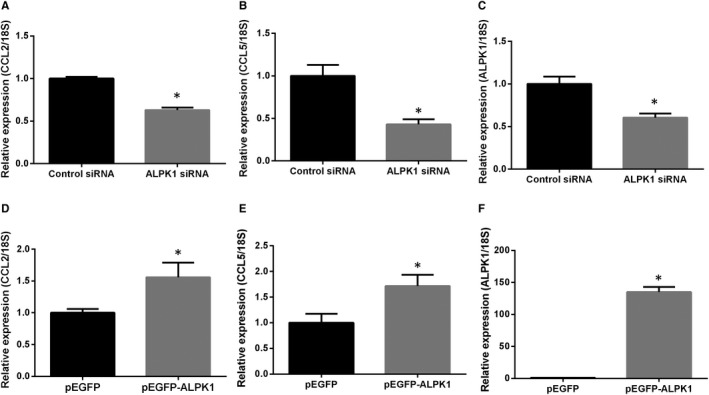
Effect of ALPK1 on CCL2 and CCL5 expression in cultured kidney cells (A‐C). HK‐2 cells were transfected with ALPK1 or control siRNA for 48h. Transcripts of ALPK1, CCL2 and CCL5 were measured by RT‐qPCR (D‐F). HEK293 cells were transfected with pEGFP‐ALPK1 or control pEGFP vectors for 48 h. ALPK1, CCL2 and CCL5 mRNA levels were measured by RT‐qPCR. Results of RT‐qPCR were repeated for three times. ∗*P* < .05 compared with cells transfected with control siRNA/pEGFP vectors.

To further elucidate the effect of ALPK1 on CCL2 and CCL5 expressions, we knock down the endogenous ALPK1 gene expression in HK‐2 cells. ALPK1 reduction decreased CCL2 and CCL5 mRNA expressions significantly (*P* < .05) (Figure 7A‐C). Endogenous ALPK1 expression is rarely shown in HEK293 cells.^9^ Therefore, we transfected pEGFP and pEGFP‐ALPK1 plasmids into HEK293 cells. The up‐regulation of CCL2 and CCL5 mRNA expression was observed in ALPK1‐overexpressed cells (Figure 7D‐F). Taken together, these results indicate positive association between ALPK1, CCL2 and CCL5 expressions.

## 4 DISCUSSION

Genetic epidemiological studies have identified ALPK1 as a susceptible gene responsible for causing gout.^3^, ^9^ Later studies indicated the associations between ALPK1 and many chronic diseases such as CKD, myocardial infarction, coronary artery disease and type 2 DM.^2^, ^21^, ^22^, ^23^, ^24^ Increasing evidence demonstrates that chronic inflammation is the major cause of these common diseases.^25^ Recent studies have demonstrated that ALPK1 is required for the phosphorylation‐dependent formation of TIFA complexes, with TRAF2 resulting in NFkB activation‐mediated secretion of inflammatory cytokines, including IL‐8, during bacterial infection.^11^, ^12^ Although the effects of MSU crystals and testosterone on TIFA complexes have not been reported, the regulation of the induction of proinflammatory cytokines might be through the TIFA pathway. Several studies have also demonstrated that high‐glucose levels can induce NFkB activation.^26^, ^27^ Therefore, regulation of the ALPK1‐TIFA‐NFkB pathway might play a predominant role in the pathogenesis of chronic diseases, in addition to its role in the cellular response against bacterial infections.

The chemokine CCL2, a member of C–C chemokine family, is ubiquitously expressed in various cell types and is responded to a wide variety of stimulation.^28^ Elevated levels of CCL2 in blood have been found in patients with type 2 DM.^28^ The CCL2 knockout mice show reduced macrophage accumulation in the kidney and the progression of renal injury in diabetic conditions.^29^, ^30^ CCL5 also belongs to the C–C chemokine family. It is secreted by various cell types including endothelial cells, smooth muscle cells, macrophages, platelets and activated T cells.^31^ High CCL5 levels in serum were measured in type 2 DM patients with hypertriglyceridaemia.^32^ CCL5 is reported to mediate acute renal injury in mice, whereas CCL5 knockout mice show better renal function during kidney ischaemia‐reperfusion injury.^33^ High levels of CCL2 were detected in tubular cells in biopsies from patients with type 2 DM and overt DN, correlating with NF‐kappaB activation in the same cells of kidney.^34^ Production of CCL5 by renal tubular cells during acute renal injury in mice is reported.^33^ We have demonstrated abundant expression of ALPK1 in renal tubular cells of mice.^6^ These shreds of evidence strongly suggest that ALPK1 enhances CCL2 and CCL5 expression through NFkB activation in hyperglycaemic conditions in renal tubular cells.

G‐CSF, a hematopoietic growth factor, showed protective effects in a variety of renal disease models including DN in rat.^35^ In the present study, the up‐regulation of G‐CSF in renal cell suffering high‐glucose stimulation might be a protective mechanism for preventing renal injury. Kidney deposition of urate crystals has been observed in patients with chronic gouty arthritis, which predisposes patients to renal fibrotic injury.^36^ The increased level of mannose‐binding lectin (MBL) regulates risk of developing microalbuminuria and DN.^37^, ^38^, ^39^ In addition, Axelgaard et al^40^ reported that MBL accumulates in the kidney during late DN. These results implicate the critical role of ALPK1 in the pathogenesis of renal injury in patients with chronic gout. NFkB pathway is activated in both human patients with kidney diseases and experimental animal models of renal inflammation and injury. Conventional therapies for DN targeting modulation of NFkB activation have been suggested and tested. Modulation of ALPK1 activation might also be a potential therapeutic target for DN.

In conclusion, high‐glucose stimulation enhances ALPK1 expression in the kidney in vitro and in vivo. Elevated ALPK1 expression up‐regulates the renal levels of CCL2 and CCL5 in cultured kidney cell and in mice model. Our data suggest that ALPK1 is a potential mediator for CCL2 and CCL5 chemokines induction involving in ALPK1‐mediated accelerated diabetic nephropathies.

## CONFLICT OF INTEREST

The authors declare that they have no conflict of interest exists.

## AUTHOR CONTRIBUTIONS

YCK conceived, designed and supervised the study. CPL and SN conducted the experiment, integrated the results and drafted the manuscript. TMK and SN analysed data and gave suggestion on experimental design. HTH and KTY involved in microscopic experiments. All the authors were involved in the discussion and reviewed the manuscript.

## Data Availability

All data generated or analysed during this study are included in this manuscript.
